# Utility of ^99m^Tc-Sestamibi SPECT/CT in the Early Localization of Metastatic Parathyroid Carcinoma

**DOI:** 10.22038/aojnmb.2017.27484.1190

**Published:** 2018

**Authors:** Patrick Earl A Fernando, Patricia A Bautista

**Affiliations:** Department of Nuclear Medicine and PET Center, St. Luke’s Medical Center, Bonifacio Global City, Taguig, Philippines

**Keywords:** Parathyroid carcinoma, SPECT/CT, ^ 99m^Tc-sestamibi

## Abstract

Parathyroid carcinoma is very rare, with only a few documented cases. Hence, metastatic lesions are infrequently documented on scintigraphic imaging. We present a case of a 63-year-old female presenting with elevated serum levels of ionized calcium and parathyroid hormone (PTH) who was referred to our department for a parathyroid scan with SPECT/CT. Parathyroid scintigraphy showed a focus of increased ^99m^Tc-sestamibi uptake corresponding to a solid mass with calcification in the inferior pole of the right thyroid lobe; tracer retention was noted on delayed images. Incidentally, focal uptake was also seen in a soft tissue mass on the 7^th^ right rib. The patient soon underwent total thyroidectomy, with biopsy revealing parathyroid carcinoma on the right lobe. A bone scan done 7 months after surgery confirmed the presence of metastatic bone disease. The concomitant detection of intrathyroidal and extrathyroidal sestamibi-avid masses on parathyroid scintigraphy should increase clinical suspicion of a metastatic process from parathyroid carcinoma.

## Introduction

Hyperparathyroidism is characterized by hypersecretion of parathyroid hormone (PTH) in the bloodstream, leading to biochemical changes such as hypercalcemia and hypercalciuria (1). Consequently, there is an enhanced bone wasting, nephrocalcinosis, and hypophosphatemia secondary to increased gastrointestinal reabsorption of calcium and 1,25-dihydroxyvitamin D_3_ (2). Hyperparathyroidism may be classified as primary, secondary, or tertiary, depending on the cause. Clinical evaluation includes screening for serum PTH, vitamin D and electrolyte levels (mainly calcium and phosphorus), among others.

Parathyroid scintigraphy using ^99m^Tc-sestamibi is an important imaging tool in the work-up of hyperparathyroidism. Its purpose is to ascertain the location of the hyperfunctioning parathyroid gland/s for possible surgical intervention (3). The advent of single-photon emission computed tomography (SPECT) and SPECT/CT has allowed for more specific pre-operative localization of hyperfunctioning parathyroid tissue.

Solitary parathyroid adenoma accounts for up to 85% of all causes of primary hyperparathyroidism. Because such an adenoma has no predilection site among the four parathyroid locations, scintigraphic imaging is important to determine its location. Surgical excision is curative for this benign condition (1).

A rare cause of primary hyperparathyroidism is parathyroid carcinoma. It is one of the most uncommon malignancies, with an estimated prevalence of 0.005% of all cancers and less than 1000 reported cases in English medical literature (4). It is usually a solitary lesion on diagnosis, with an indolent but slowly progressive course; local recurrence and metastases are frequent. Radical excision, along with the ipsilateral thyroid lobe, remains the standard of care (5).

A parathyroid scan alone cannot distinguish if a lesion is benign or malignant. However, its diagnostic value is in revealing the presence of ectopic and abnormal parathyroid tissue in the anterior neck and elsewhere (6). The presence of a sestamibi-avid lesion outside of the anterior neck raises the suspicion for a metastatic process.

There have been studies assessing the utility of ^99m^Tc-sestamibi scintigraphy in establishing parathyroid carcinoma. However, findings have been mostly divergent. It was found to have a sensitivity of 91%, and in one case report an intrathoracic parathyroid carcinoma was localized using a ^99m^Tc-sestamibi scan with SPECT/CT (5). Yet a separate case series noted that it failed to localize mediastinal recurrence and pulmonary metastasis in one case (7). Another case report cited a positive histopathologic report of metastatic bone disease from parathyroid carcinoma despite negative ^99m^Tc-sestamibi, ^99m^Tc-MDP and ^18^F-FDG PET scans (8). These findings may indicate that ^99m^Tc-sestamibi scintigraphy in parathyroid carcinoma is more sensitive in detecting ectopic sites of parathyroid carcinoma rather than recurrences of such. 

## Case report

A 63-year-old female was admitted at our institution due to generalized body weakness and anorexia. She is a known hypertensive with chronic kidney disease. Upon further work-up, she was assessed to have severe hyponatremia (116 mmol/L), hypokalemia (2.8 mmol/L) and hypomagnesemia (1.2 mg/dL). Ionized calcium was 1.82 mmol/L (reference range: 1.00–1.30 mmol/L), serum PTH was 3071.90 pg/mL (reference range: 14.0–72.0 pg/mL), and serum vitamin D was 35.52 ng/mL (reference range: ≥ 30 ng/mL). Because of the clinical suspicion for primary hyperparathyroidism, a parathyroid scan was requested for further evaluation.

**Figure 1:**
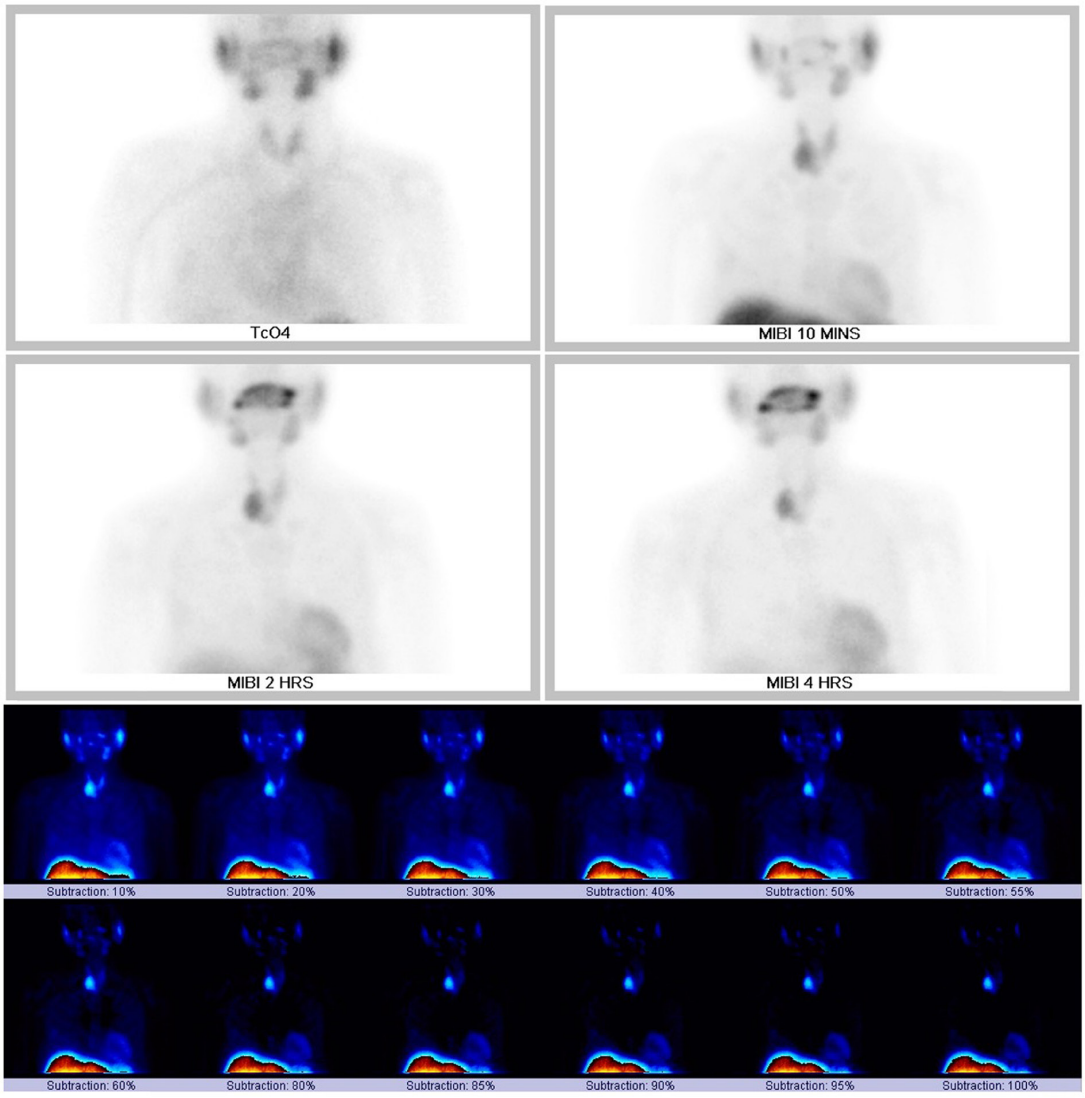
Parathyroid scintigraphy showed focal uptake of ^99m^Tc-sestamibi in the inferior pole of the right thyroid lobe on initial (10 minutes), delayed (2 and 4 hours), and subtraction images

**Figure 2:**
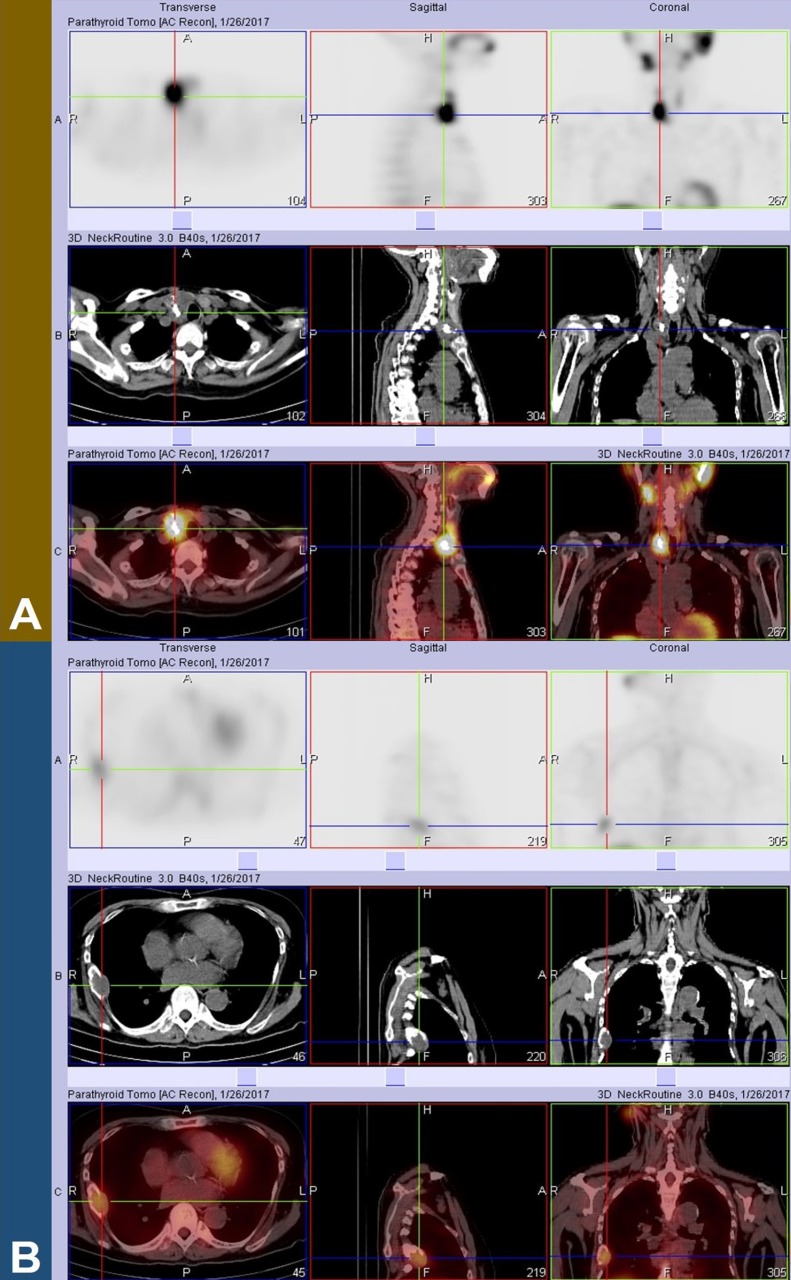
SPECT/CT revealed a sestamibi-avid lesion in the inferior pole of the right lobe (A), corresponding to a solid mass with calcification. An incidental focus of increased tracer uptake was also noted in a soft tissue mass on the 7^th^ right rib (B

**Figure 3:**
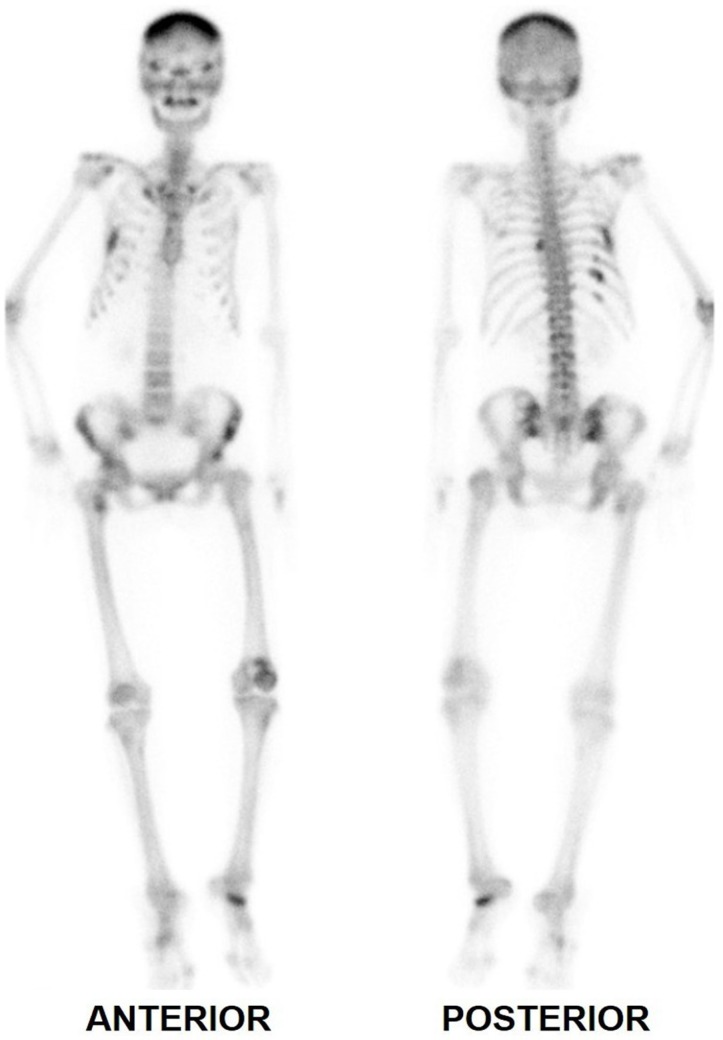
Bone scintigraphy 7 months after total thyroidectomy showed multiple lesions in the axial and appendicular skeleton that appear metastatic in nature

**Figure 4:**
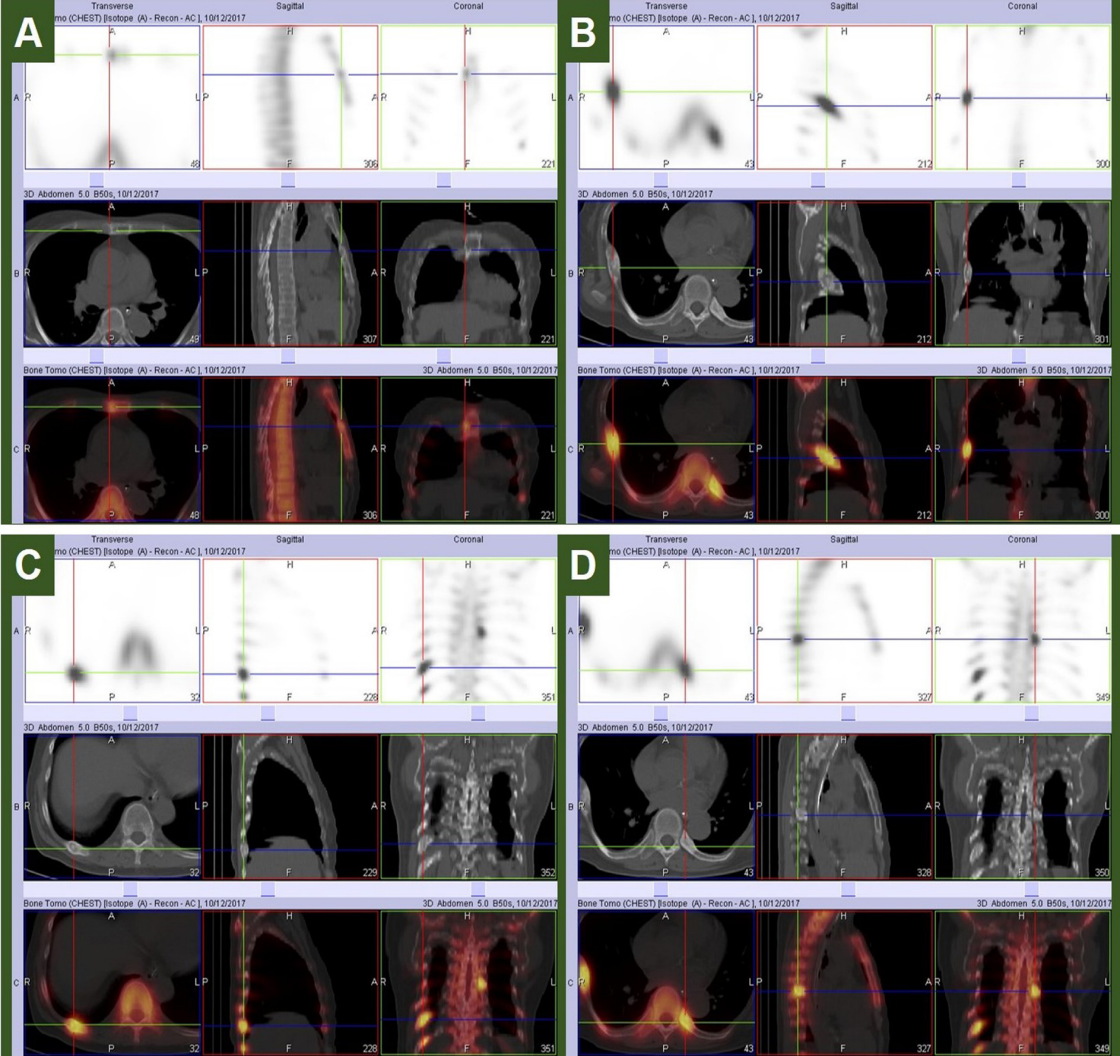
SPECT/CT revealed multiple MDP-avid metastatic foci: (A) mild focus on the right side of the sternum showing mild bone erosion; (B) soft tissue mass on the 7^th^ right rib that was also sestamibi-avid on parathyroid scan; (C) posterior aspect on the 10^th^ right rib showing another soft tissue mass; and (D) 8^th^ left costovertebral junction

***Parathyroid Scintigraphy with SPECT/CT***

Parathyroid scintigraphy was performed using a dual-tracer protocol and with SPECT/CT as per institutional standard procedure. Planar imaging of the neck and thorax was done 15 minutes after intravenous injection of 173.9 MBq of ^99m^Tc-pertechnetate. Planar and SPECT/CT images of the neck and thorax were acquired 15 minutes after injection of 769.6 MBq of ^99m^Tc-sestamibi. Delayed 2- and 4-hour planar images were also obtained.

Static and subtraction images are illustrated in Figure 1. The ^99m^Tc-pertechnetate image showed decreased tracer uptake in the inferior half of the right thyroid lobe but fairly homogeneous tracer uptake in the left lobe. The initial ^99m^Tc-sestamibi planar image revealed a large focus of increased tracer activity in the inferior pole of the right lobe. The said focus showed retained activity on subtraction images and was found to arise from a solid mass with calcification on SPECT/CT (Figure 2A). Incidentally, a fainter focus of tracer uptake was noted in a soft tissue mass on the lateral aspect of the 7^th^ right rib (Figure 2B). 

Delayed 2- and 4-hour planar images (Figure 1) showed tracer retention in the inferior pole of the right lobe. Symmetric tracer clearance was seen in the rest of the thyroid parenchyma. Physiologic tracer uptake was seen in the naso- and oropharynx, salivary glands, myocardium and liver dome.

Histopathologic correlation was recommended for the sestamibi-avid mass with calcification in the inferior pole of the right thyroid lobe. This was attributed to a parathyroid carcinoma or adenoma, although a thyroid pathology was also possible. A similar recommendation was made for the incidental sestamibi-avid soft tissue mass in the lateral aspect of the 7^th^ right rib, as it was highly suspicious for metastasis.

The patient was subsequently given intravenous furosemide for calcium correction. Intravenous saline solution was titrated for sodium correction. Intravenous potassium chloride, then later oral potassium citrate, was given for potassium correction. Magnesium sulfate infusion was given to correct for magnesium. After 10 days of admission, the patient was deemed to have acceptable electrolyte levels (ionized calcium 1.39 mmol/L; sodium 136.9 mmol/L; potassium 4.4 mmol/L; magnesium 2.2 mg/dL) and no subjective complaints. She was thus discharged and started on Cinacalcet, a calcimimetic drug, to address the patient’s hyperparathyroid state.

***Surgery and Histopathology***

She underwent total thyroidectomy in another institution two months post-discharge. The final histopathologic diagnosis was as follows:

- Favor a parathyroid carcinoma, right lobe. Lymphovascular space invasion seen. Negative for tumor: surgical resection margins. Recommend immunohistochemical staining.
- Papillary microcarcinoma (0.2 cm greatest diameter), limited to the left lobe. Negative for extrathyroidal extension.
- Multinodular colloid goiter with hyperplastic features, isthmus and pyramidal lobe.

Histopathologic slide review was done in our institution 7 months later. The final assessment showed an enlarged hypercellular parathyroid consistent with parathyroid carcinoma. The pathologist remarked that while the lesion is not mitotically active nor is there the presence of more recognizable definitive criteria for malignancy such as vascular and perineural invasion in the submitted material, the lesion shows extension beyond the capsule and encroaching on sizable extraglandular vessels.

Immunohistochemical staining was also performed on slide review and was said to substantiate the above findings. The specimen was positive for chromogranin and negative for synaptophysin, thyroglobulin and calcitonin. Ki-67 was found to be less than 5% (low index of proliferation).

***Metastatic Work-Up: Bone Scan***

The patient was lost to follow-up after surgery. Seven months later, she was re-admitted at our institution due to alleged behavioral changes. Serum sodium, potassium and calcium were within normal limits. Repeat PTH was noted to be 438.70 pg/mL. Once she was stabilized, she was referred to the Medical Oncology service for possible chemotherapy initiation. A bone scan was subsequently requested for metastatic work-up.

Whole body bone scintigraphy in the anterior and posterior projections, with additional views of the head, thorax and pelvis, was performed 2.5 hours after intravenous injection of 1054.5 MBq of ^99m^Tc-methylene diphosphonate (MDP). SPECT/CT of the head and chest was also done.

Planar and SPECT/CT images are shown in Figures 3 and 4, respectively. The kidneys appeared faint on planar images. Foci of increased tracer uptake were noted in the following areas:

- right side of the sternum (mild focus corresponding to mild bone erosion)
- 4^th^ left rib
- 7^th^ right rib (corresponding to a soft tissue mass on SPECT/CT, previously noted to be sestamibi-avid on parathyroid scintigraphy)
- 9^th^ to 11^th^ right ribs (uptake in the 10^th^ rib corresponding to another soft tissue mass)
- 8^th^ left costovertebral junction
- sacroiliac junctions (photopenic foci surrounded by a tracer-avid rim) and left iliac bone
- right lesser trochanter

The above findings were assessed to be consistent with metastatic bone disease.

## Discussion

This report focuses on a female patient with electrolyte imbalance who was noted to have elevated PTH and ionized calcium levels. Parathyroid scintigraphy not only confirmed the clinical suspicion of primary hyperparathyroidism but also gave a hint that a metastatic carcinoma may be present. This was eventually confirmed by the post-thyroidectomy biopsy report, as well as the bone scan findings on follow-up.

Because parathyroid carcinoma is very uncommon, little can be said regarding its behavior in metastatic disease: its predilection sites of tumor spread and the type of lesions it produces when bone is involved (osteoblastic, osteolytic or mixed). For the same reason, the clinical tendency is to address a positive parathyroid scan as an adenoma, which is significantly more frequent. 

In retrospect, the case exhibited several features that favored a diagnosis of parathyroid carcinoma. In terms of serology, the patient’s PTH level was 42 times the upper limit reference range. Benign parathyroid tumors cause a milder elevation of PTH than in malignant disease, where up to a 75-fold elevation may be seen (6).

Majority of parathyroid carcinomas present with functioning tumors (9). In the case of the patient, there is an incidental finding of a sestamibi-avid soft tissue lesion on the 7^th^ right rib during parathyroid scintigraphy. The patient had no symptoms relating to this lesion at the time of the scan. This supported the possibility of a functioning parathyroid tumor outside of the anterior neck. A differential diagnosis is that it may have been a brown tumor: a focal, benign bony lesion caused by rapid bone turnover as a direct effect of primary hyperparathyroidism. Like parathyroid carcinoma, brown tumors are uncommon, seen only in less than 5% of hyperparathyroid patients. Common clinical features include bone pains, swelling and pathologic fractures, none of which was clinically manifested by the patient in this case (10).

It was unfortunate that the patient was lost to follow-up for several months after surgery, as initiation of therapy could have been done earlier. Bone scintigraphy eventually confirmed the suspicion of metastasis seen on parathyroid scan. It was noted that the sestamibi-avid rib lesion was also MDP-avid, indicating increased osteoblastic activity. The pattern of distribution of these osteoblastic lesions, as well as another soft tissue mass seen on the 10^th^ right rib, were consistent with metastatic parathyroid carcinoma.

When compared with parathyroid scintigraphy, there were more lesions noted on bone scan. This is consistent with multiple osseous metastases, as areas of increased osteoblastic activity tend to be MDP-avid. In contrast, less sestamibi-avid lesions were seen on parathyroid scan. This implies the presence of ectopic parathyroid tissue and is supportive of a carcinoma of parathyroid origin.

The presence of non-osseous metastatic lesions cannot be ruled out in this case. As such, further diagnostic work-up may be suggested. A ^18^F-FDG PET scan may be prudent at this point, as it is able to provide additional information on the extent of parathyroid carcinoma compared to CT, MRI and ^99m^Tc-sestamibi. In addition, this scan may be used as a baseline reference for follow-up studies that may be done after treatment (11).

This case report highlights the utility of ^99m^Tc-sestamibi parathyroid scintigraphy with SPECT/CT in the early recognition of the possibility of metastatic disease. The detection of intrathyroidal and extrathyroidal lesions on parathyroid scan should increase the clinical suspicion of an aggressive process, such as parathyroid carcinoma. For similar cases in the future, bone scintigraphy 2-3 days after the parathyroid scan is suggested to further evaluate the whole body for osseous metastasis.
